# Seasonal Variation of Testosterone Levels in a Large Cohort of Men

**DOI:** 10.1155/2022/6093092

**Published:** 2022-06-22

**Authors:** Taiba Zornitzki, Sagi Tshori, Galit Shefer, Shira Mingelgrin, Carmit Levy, Hilla Knobler

**Affiliations:** ^1^Diabetes, Endocrinology and Metabolic Disease Institute, Kaplan Medical Center, Rehovot, Israel; ^2^Hebrew University, The Faculty of Medicine, Jerusalem, Israel; ^3^Research Authority, Kaplan Medical Center, Rehovot, Israel; ^4^Department of Human Genetics and Biochemistry, Sackler Faculty of Medicine, Tel Aviv University, Tel Aviv, Israel

## Abstract

**Objectives:**

The aim of the study was to evaluate in a large cohort of males with a wide range of age, metabolic status, and coexistent morbidities whether month of blood test performance was associated with total and bioavailable testosterone levels independent of age, body mass index (BMI), existing cardiovascular disease (CVD), and CVD risk factors.

**Methods:**

Cross-sectional study includes data from computerized medical records of 27,328 men aged 20–70, treated by the largest healthcare organization in Israel, who had undergone testosterone measurement. In 7,940 subjects with available sex-hormone-binding globulin levels, bioavailable testosterone was calculated.

**Results:**

Total and bioavailable testosterone levels gradually decreased with age and BMI (*P* < 0.001) and were significantly lower in men with diabetes, hypertension, hyperlipidemia, and known CVD, but were higher in current smokers compared with nonsmokers (*P* < 0.001). Hormone levels were highest in August-October declined after and lowest in March. Overall, both total and bioavailable testosterone levels were significantly lower in March compared to August-October (*P* < 0.001). In a linear regression analysis, age, BMI, current smoking, and month of testing were independently associated with total (*P* < 0.001) and bioavailable testosterone levels (*P*=0.002), and diabetes was associated with total testosterone (*P* < 0.001).

**Conclusion:**

In a large cohort of men with a wide range of age, BMI, and comorbidities, month of testing was independently associated with total and bioavailable testosterone levels. These data provide strong evidence that seasonal variation has to be considered in clinical practice.

## 1. Introduction

Numerous studies have shown the testosterone levels decrease with aging, obesity, and other features of the metabolic syndrome. The decrease in testosterone levels in these clinical settings has been implicated to have an adverse effect on male health status including increased cardiovascular disease (CVD) risk [1, 2]. Several studies also demonstrated that testosterone treatment led to improvement in the metabolic syndrome features and CVD risk factors. However, this therapeutic approach is still debated [3–6].

Diurnal variation in testosterone levels is well recognized. Testosterone levels are relatively high in the morning and decline modestly in the evening [7]. Therefore, clinical practice guidelines recommend measuring testosterone levels in the early morning hours [8].

However, there are several human studies suggesting that there is also seasonal variation in testosterone levels [9–15]. This intriguing phenomenon is supported by animal data demonstrating that the hypothalamic-pituitary-gonadal axis is susceptible to several environmental conditions. One of these stimuli is exposure to ultraviolet (UV) light [16]. In our recent study, we explored a novel skin-brain-gonad axis triggered by UV light that is mediated by skin p53. By using various mouse models, we found that chronic UV light exposure led in male and female mice, to increased pituitary and gonadal hormone levels and to increased sexual responsiveness and attractiveness [17].

Therefore, the following question arises: do we have enough evidence to support the need of including seasonal testosterone variation in our treatment decisions? Up to now, this notion has not gained much attention due to conflicting results derived from human studies [13, 18–20]. Previous studies were limited by either small sample size or variability in baseline characteristics and confounders, such as differences in age and weight distribution, coexistent illness, time in the day in which testosterone was measured, and also by differences in geographic location and climate conditions.

The aim of the current study was to evaluate the relationship between testosterone levels and month of testosterone determination in a large cohort of Israeli males aged 20–70 years old with a wide range of coexistent morbidities, after adjusting for age, body mass index (BMI), existing CVD, and CVD risk factors. In subjects with available sex-hormone-binding globulin (SHBG) levels, we determined the relationship between bioavailable testosterone and all of the above parameters.

## 2. Methods

### 2.1. Study Design and Database

All Israeli citizens are entitled to a basic healthcare insurance from one of four health maintenance organizations (HMOs). Clalit Health Services (CHS) is the largest not-for-profit HMO in Israel and one of the largest HMOs in the world, covering around 53% of the Israeli population and providing an extensive network of hospitals, and primary and specialized clinics. CHS insures members of all ages, ethnic groups, and socioeconomic backgrounds across the country. CHS and other health funds in Israel are characterized by a particularly low annual turnover of about 1%, which enables a long follow-up period.

In this cross-sectional study, we retrieved records of 43,244 men aged 20–70 years, members of CHS in Jerusalem and central districts, who performed blood testosterone testing between 2009 and 2019. Data were retrieved from the Clalit Secure Data Sharing Platform powered by MDClone (https://www.mdclone.com). Blood drawing in all CHS clinics is routinely performed in the morning hours between 7 AM and 9 AM.

Exclusion criteria included malignancy, acute or chronic renal failure, epilepsy, psychiatric disorders, uncontrolled endocrine disorders, testicular or male infertility disorders, and treatment by aldosterone antagonists, ketoconazole or high-dose steroids (higher than 5 mg prednisone or equivalent dose of other steroid daily) during the 12 months prior to testosterone measurement. Finally, 27,328 men were included in the study.

For each subject, the following data were extracted: age, body mass index (BMI), smoking status, country of birth, prescriptions, diagnoses including CVD, and chronic CVD risk factors: diabetes mellitus (E08.1, E08.10, E08.11, E08.42, E08.9, E10.10, E10.11, E10.29, E10.65, E10.8, E11.00, E11.21, E11.29, E11.311, E11.40, E11.51, E11.618, E11.641, E11.65, E11.9, E13, E13.3, E13.31, E13.311, E13.36, E13.4, E13.5, E13.69, E13.9, Z83.3), ischemic heart disease (I24, I24.8, I25.1, I25.10, I25.2, I25.3, I25.5, I25.8, I25.810, I25.811, I25.82, I25.83, I25.84, I25.9), hypertension (I10, I11.0, I11.9, I12.0, I13, I15, I15.0, I15.8), and hyperlipidemia (E78, E78.0, E78.2, E78.3, E78.4, E78.6, E78.89, E78.9).

### 2.2. Laboratory Data

Testosterone tests were performed in a number of CHS-certified laboratories using standardized automated methods. Therefore, testosterone values have been adjusted to the results of ADVIA Centaur TSTII assay (Siemens) performed in the central laboratory, using the method described by Karvanen [21]. Normalization was performed according to the location-scale model, assuring the results are centered around the same value. This method is preferred to classic normalization, using mean and standard deviation, because it allows to refrain from further assumptions. Each value was transformed based on the reference limits at its facility, to achieve standardized value. The standardized value was calculated by subtracting the lower reference limit and then dividing by the reference range. The standardized values were later adjusted using the central laboratory reference limits, multiplying by the range and adding the lower reference limit. The lower and upper normal limits used in the central laboratory were 8.4 and 28.7 nmol/L, respectively.

SHBG tests performed in the two years before or after the testosterone test were available in 7,940 subjects. Bioavailable testosterone was calculated using the method described by Vermeulen et al. [22], with the assumption of an average serum albumin concentration of 4.3 mg/dL.

### 2.3. Statistical Analysis

Continuous variables were evaluated for normal distribution using histogram and Q-Q plot. Descriptive statistics were used to describe the distribution of total and bioavailable testosterone by age, BMI, CVD, and cardiovascular risk factors. Significance level of the effect on testosterone was estimated using univariate analysis of variance. Results are presented as mean ± SD unless otherwise specified.

The difference in testosterone levels between March and August-October was estimated using univariate analysis and Welch's *t*-test. In addition, Tukey's range test was used for pairwise comparison, adjusted for variables found statistically significant in the multivariate analysis of variance. Multiple linear regressions were used to predict the total and the bioavailable testosterone levels, with the following variables: age, BMI, smoking, diabetes, CVD, hypertension, hyperlipidemia, and month of blood testosterone testing.

All statistical tests were 2-sided. *P* value <0.05 was defined as statistically significant. Data were analyzed using R version 3.6.3.

## 3. Results

### 3.1. The Effect of Age, BMI, CVD, and CVD Risk Factors on Total Testosterone and Bioavailable Testosterone Levels

A total of 27,328 men aged 20–70 were included in the study. Total testosterone levels in the population were stratified into groups based on age, BMI, known CVD, and CVD risk factors: smoking status, diabetes, hypertension, and hyperlipidemia (Table 1). Testosterone levels gradually decreased with age, being highest in the 20–25 years age group and lowest in the 65–70 years age group (17.6 ± 6.1 nmol/L vs. 14.2 ± 5.3 nmol/L, *P* < 0.001). Testosterone levels were inversely associated with BMI, being highest in the lowest BMI group (<18.5 kg/m^2^) and lowest in the highest BMI (>40 kg/m^2^) (19.4 ± 6.2 nmol/L vs. 10.3 ± 4.2 nmol/L, *P* < 0.001).

Since previous data suggest that men with CVD or CVD risk factors have lower testosterone levels, we assessed the effect of these conditions on testosterone levels in our cohort. Total testosterone levels were significantly lower in men with the following illness compared with those without these conditions: diabetes (13.2 ± 5.2 nmol/L vs. 15.5 ± 5.7 nmol/L, *P* < 0.001), hypertension (13.4 ± 5.0 nmol/L vs. 15.6 ± 5.8 nmol/L, *P* < 0.001), and hyperlipidemia (14.1 ± 5.2 nmol/L vs. 15.5 ± 5.8 nmol/L, *P* < 0.001). Current smoking was associated with higher levels of total testosterone compared with nonsmokers and past smokers (16.0 ± 6.0 nmol/L vs. 15.1 ± 5.6 nmol/L and 14.4 ± 5.4, respectively, *P* < 0.001). Testosterone levels were significantly lower in known CVD patients compared to men without known CVD (13.5 ± 5.1 nmol/L vs. 15.3 ± 5.7 nmol/L, *P* < 0.001).

SHBG levels were available in 7,490 subjects, allowing the calculation of bioavailable testosterone (Table 2). Similar to total testosterone levels, bioavailable testosterone levels gradually decreased with age, being highest in the 20–25 years age group and lowest in the 65–70 years age group (9.0 ± 3.2 nmol/L vs. 5.6 ± 1.9 nmol/L, *P* < 0.001). Also, bioavailable testosterone levels were inversely associated with BMI, being highest in the low BMI group (<18.5 kg/m^2^) and lowest in the high BMI group (>40 kg/m^2^) (8.0 ± 3.3 nmol/L vs. 5.4 ± 2.2 nmol/L, *P* < 0.001). Diabetes and hypertension were associated with lower bioavailable testosterone levels. Current smoking was associated with higher bioavailable testosterone levels (7.2 ± 2.7 nmol/L vs. 7.1 ± 2.7 nmol/L and 6.4 ± 2.4 in nonsmokers and past smokers, *P* < 0.001). Bioavailable testosterone levels were lower in the CVD group compared to males without known CVD (5.8 ± 2.0 nmol/L vs. 7.1 ± 2.7 nmol/L, *P* < 0.001).

### 3.2. Seasonal Variation in Testosterone Levels

**Figure 1:**
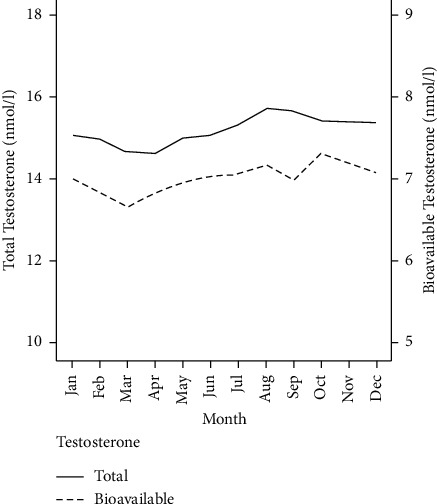
Monthly variations in total and bioavailable testosterone levels in a cohort of men aged 20–70.

In order to assess whether there is seasonal variation in testosterone levels, we stratified total and bioavailable testosterone levels according to the month in which the test was performed (Figure 1). A peak of total testosterone levels was observed from August to October (15.7 ± 5.9 nmol/L, 15.7 ± 5.9, and 15.4 ± 5.8 nmol/L, respectively). Similarly, a peak from August to October was observed for bioavailable testosterone (7.2 ± 2.8 nmol/L, 7.0 ± 2.8, and 7.3 ± 3.0 nmol/L, respectively). After October, total testosterone and bioavailable testosterone started to decline reaching a nadir in March (14.7 ± 5.6 nmol/L and 6.7 ± 2.5 nmol/L, respectively). Overall, both total testosterone and bioavailable testosterone levels were significantly lower in March compared to the levels measured in August-October (*P* < 0.001) (Table 3).

### 3.3. Linear Regression Analysis

Linear regression analyses were performed (Table 4). The confounders' age, BMI, and the variables current smoking (but not past smoking) and diabetes were independently associated with total testosterone (*P* < 0.001). The same variables were also independently associated with bioavailable testosterone levels.

Importantly, the month of the year in which hormone levels were performed was independently associated with total testosterone (*P* < 0.001) and with bioavailable testosterone levels (*p*=0.0013).

## 4. Discussion

In the current study that included a large cohort of men with a wide range of age groups, BMI, and comorbidities, we found a significant association between the time in the year in which blood test was performed and both total and bioavailable testosterone levels. Furthermore, the month in which testosterone was measured was independently associated with its level in a linear regression analysis that included other baseline characteristics that were found to affect this hormone levels: age, BMI, diabetes, and current smoking. A similar finding was observed for bioavailable testosterone. Our data show that total and bioavailable testosterone levels reach peak levels in August-October (summer-autumn period) and afterward decline with a nadir in March (winter). Due to the large size of our cohort, this study provides strong supporting evidence to the notion of seasonal variation in testosterone levels. In addition, this study confirms previous studies showing that testosterone levels are also reduced with age, increasing BMI, CVD, and CVD risk factors except current smoking [23, 24].

Intriguingly, the effect of exposure to UV light on testosterone levels in males was already described almost a century ago in 1939 [25]. UV light is one of several environmental stimuli that can affect the circadian rhythm with its important regulatory role in reproduction [26]. In our recently published study, we describe a novel mechanism underlying a skin-brain-gonad axis [17]. Chronic UV exposure led to increased levels of sex hormones in male and female mice and increased sexual responsiveness and attractiveness. Conditional knockout of p53 specifically in skin keratinocytes abolished the effects of UV light. Thus, UV triggers a skin-brain-gonadal axis through skin p53 activation [17].

Several studies within the last years provided conflicting results regarding the existence of seasonal variation in testosterone levels in humans [12, 13, 18–20]. The discrepancy between studies can be explained by the variability in baseline characteristics and confounders. One important factor that has to be taken into consideration is the geographic location of each conducted study, which affects the seasons' length, temperature variation, and day-night cycles. In Israel, winter starts at about November and lasts until the end of March and the temperature in most parts of the country is moderately cold and rainy. In April, the temperature rises gradually, and from June until about the mid of September, the weather becomes hot without rain. Therefore, our data demonstrate higher testosterone levels during the summer-autumn season and nadir in the winter.

These data confirm the results of a previous study conducted in Italy, another Mediterranean country, including 7,491 men demonstrating seasonal variation of testosterone with the highest levels in summer time [13]. Similar pattern was observed in two studies from Europe and USA [10, 14]. The peak of testosterone levels in summer correlates with longer daylight duration and higher temperature [13]. Testosterone levels can also be affected by a seasonal change in melatonin by-product levels and sleep-wake pattern [12]. Another potential season-dependent change is the degree of physical activity, since generally there is a significant increase in physical activity during the summer months [27]. Strenuous physical activity has been shown to be a potent stimulus of testosterone production [28].

However, other previous studies provided differing results. In a study conducted in Norway including 1,548 men, Svartberg et al. showed a bimodal seasonal variation of total testosterone with a small peak in February, a nadir in June, and a more prominent peak in October and November [9]. In a Korean population, a nadir of testosterone levels was observed in May and a peak in January [15]. Other studies have not identified seasonal variability in testosterone levels [18–20]. These dissimilarities can be explained in part by differences in climate between countries.

Testosterone levels were shown by numerous studies to be affected significantly by other factors such as age, BMI, and coexistent medical conditions [23, 24]. Our data confirm those previous observations. Therefore, the effect of seasonal variation in testosterone levels cannot be determined without taking into account those variables. The strength of our study is including a large unselected group of men, with a wide range of age, BMI, and comorbidities. This design enabled us to analyze the effect of different baseline characteristics besides season, on testosterone levels. In contrast, some previous studies included a more selected cohort. For example, the study of Lee and Lee included individuals who were part of police officer urological health screening program [15] and another study included mainly older men or those with high total cholesterol and/or low high-density lipoprotein cholesterol levels [9]. Differences in these baseline characteristics may affect testosterone levels irrespective of environmental conditions.

Intriguingly, in the study of Santi et al., seasonal changes in luteinizing hormone and testosterone levels were found only in the age group between 35 to 57 years [13]. Two previous studies conducted in Europe and USA that demonstrated seasonal variation in testosterone levels included relatively young men [10, 14]. Blunting of the 24-h variation in testosterone levels in elderly men has been shown in the past [7, 29], and further studies are needed to clarify whether aging also affects seasonal testosterone variation. The significant effect of aging on testosterone levels was confirmed by our study. Both total and bioavailable testosterone levels gradually decreased with age, being highest in the 20–25 years age group and lowest in the 65–70 years age group. Aging is also associated with an increase in obesity and metabolic disturbances, which were shown to affect testosterone levels [30, 31]. Our data conform the significant effect of obesity demonstrating that both total and bioavailable testosterone levels were inversely associated with BMI, being highest with low BMI and lowest with high BMI. Regarding other comorbidities that are part of the metabolic syndrome, namely, diabetes, hypertension, hyperlipidemia, and cardiovascular diseases, we found that men with those conditions had lower total and bioavailable testosterone levels.

Interestingly, although current smoking is a strong CVD risk factor, it was associated with higher levels of total and bioavailable testosterone compared with those who never smoked or smoked in the past. This finding confirms few previous data [23, 32, 33]. Furthermore, both total and free testosterones increased gradually with the number of cigarettes smoked daily. This effect may be explained by the nicotine metabolite cotinine that can inhibit androgen breakdown or be related to some personality differences between nonsmokers and smokers [32]. Therefore, smoking could possibly mask borderline hypogonadism and is an important confounding factor that cannot be ignored in studies.

### 4.1. Limitations and Advantages of the Study

The main advantage of the study is that included a large cohort of men who are treated by the largest HMO in Israel that provides care to a diverse population including all age groups, ethnic origin, socioeconomic backgrounds, metabolic status, and various comorbidities. CHS computerized system includes a comprehensive record of demographic, anthropometric, medical diagnosis, medications, and laboratory data. Therefore, this enabled determining the effect of seasonal variation in testosterone levels in a population with a wide range of baseline characteristics and adjusting for age, BMI, and coexistent illnesses that can affect hormonal status. In addition, blood sampling in CHS is routinely conducted in early morning hours.

The main limitation of our study is the cross-sectional design and that hormone levels were determined at one time point. Future longitudinal large prospective studies conducted with serial testosterone measurements obtained from each single participant during the year would provide further support to the notion of seasonal variation in testosterone levels.

As discussed above, testosterone levels are affected by many environmental and geographic variables. In addition, other factors such as vitamin D status [34] and skin color that vary between different populations may modify the seasonal variability of testosterone levels. Therefore, these findings have to be replicated in other parts of the world.

Another limitation is that SHBG levels were not available for all participants. However, in general the findings based on total testosterone levels were very similar to the findings based on bioavailable testosterone levels.

## 5. Conclusion

Our study demonstrated a significant seasonal variation of total and bioavailable testosterone levels independent of age, BMI, and existence of CVD and CVD risk factors in a large cohort of males. Testosterone levels were highest in summer-autumn and lowest in winter. These findings imply that timing of testosterone determination has to be taken into account, especially in men with borderline levels. In such cases, we recommend repeating the measurement in summer-autumn season as part of treatments' decision. However, since this effect is also influenced by specific environmental conditions in different parts of the world, further studies are needed to determine geographic-specific effect.

## Figures and Tables

**Table 1:** Total testosterone levels stratified by age, BMI, smoking status, diabetes, CVD, hypertension, and hyperlipidemia.

	*N*	Mean	Median	Range	*P* value
All	27328	15.2 ± 5.7	14.5	3.6–37.0	
Age					
20–25	2568	17.6 ± 6.1	17.1	3.6–37.0	<0.001
25–30	4016	16.4 ± 5.9	15.7	3.6–37.0	
30–35	3981	15.5 ± 5.6	14.7	4.0–36.8	
35–40	2896	14.9 ± 5.6	14.2	3.6–36.7	
40–45	2227	14.9 ± 5.8	14.1	3.6–36.3	
45–50	2059	14.5 ± 5.5	13.8	3.8–36.7	
50–55	2375	14.3 ± 5.3	13.7	3.6–35.8	
55–60	2711	14.1 ± 5.3	13.3	3.6–36.9	
60–65	2557	14.3 ± 5.3	13.6	4.0–36.1	
65–70	1938	14.2 ± 5.3	13.3	3.7–36.8	
BMI (kg/m^2^)					
(<18.5)	388	19.4 ± 6.2	19.0	4.4–35.9	<0.001
(18.5, 25)	9879	17.4 ± 5.8	16.8	3.6–37.0	
(25, 30)	10851	14.6 ± 5.2	14.0	3.6–36.9	
(30, 35)	4375	12.8 ± 4.8	12.0	3.6–36.7	
(35, 40)	1203	11.4 ± 4.3	10.5	3.6–33.3	
(>40)	459	10.3 ± 4.2	9.4	3.6–36.1	
Unknown	173	15.0 ± 6.1	13.6	5.4–32.3	
Smoking status					
Never	16413	15.1 ± 5.6	14.3	3.6–36.9	<0.001
Now	6544	16.0 ± 6.0	15.3	3.6–37.0	
Past	3784	14.4 ± 5.4	13.6	3.6–36.7	
Unknown	587	14.9 ± 5.6	14.3	4.3–35.7	
Diabetes					
No	23639	15.5 ± 5.7	14.8	3.6–37.0	<0.001
Yes	3689	13.2 ± 5.2	12.2	3.6–36.8	
CVD					
No	25709	15.3 ± 5.7	14.6	3.6–37.0	<0.001
Yes	1619	13.5 ± 5.1	13.0	3.6–36.8	
Hypertension					
No	22090	15.6 ± 5.8	14.9	3.6–37.0	<0.001
Yes	5238	13.4 ± 5.0	12.6	3.6–36.8	
Hyperlipidemia					
No	21261	15.5 ± 5.8	14.8	3.6–37.0	<0.001
Yes	6067	14.1 ± 5.2	13.2	3.7–36.8	

Testosterone levels are expressed in nmol/L.

**Table 2:** Bioavailable testosterone levels stratified by age, BMI, smoking status, diabetes, CVD, hypertension, and hyperlipidemia.

	*N*	Mean	Median	Range	*P* value
All	7940	7.0 ± 2.7	6.6	0.7–30.2	
Age					
20–25	701	9.0 ± 3.2	8.5	1.0–23.1	<0.001
25–30	1190	8.1 ± 3.0	7.6	1.6–30.2	
30–35	1279	7.4 ± 2.6	7.0	1.3–18.2	
35–40	827	7.0 ± 2.4	6.7	1.8–17.7	
40–45	606	7.0 ± 2.6	6.5	2.4–25.6	
45–50	578	6.8 ± 2.3	6.4	1.5–16.8	
50–55	675	6.3 ± 2.1	6.0	1.5–13.1	
55–60	773	6.0 ± 2.1	5.6	0.7–18.7	
60–65	740	5.9 ± 1.9	5.5	1.3–13.2	
65–70	571	5.6 ± 1.9	5.4	1.2–14.5	
BMI (kg/m^2^)					
(<18.5)	122	8.0 ± 3.3	7.3	1.2–17.5	<0.001
(18.5, 25)	2892	7.7 ± 2.8	7.4	0.7–30.2	
(25, 30)	3146	6.8 ± 2.6	6.4	1.4–28.7	
(30, 35)	1240	6.2 ± 2.3	5.8	1.3–17.7	
(35, 40)	361	5.7 ± 2.0	5.2	2.2–12.6	
(>40)	124	5.4 ± 2.2	4.9	2.0–15.9	
Unknown	55	7.5 ± 2.8	6.8	3.2–16.1	
Smoking status					
Never	4829	7.1 ± 2.7	6.6	1.0–30.2	<0.001
Now	1774	7.2 ± 2.7	6.9	0.7–25.6	
Past	1113	6.4 ± 2.4	6.0	1.6–20.4	
Unknown	224	7.1 ± 2.6	6.8	2.3–14.1	
Diabetes					
No	6864	7.2 ± 2.7	6.8	1.0–30.2	<0.001
Yes	1076	6.0 ± 2.1	5.6	0.7–18.7	
CVD					
No	7471	7.1 ± 2.7	6.7	0.7–30.2	<0.001
Yes	469	5.8 ± 2.0	5.5	1.3–14.5	
Hypertension					
No	6378	7.3 ± 2.7	6.9	0.7–30.2	<0.001
Yes	1562	6.0 ± 2.1	5.6	1.3–18.7	
Hyperlipidemia					
No	6235	7.2 ± 2.7	6.8	0.7–30.2	<0.001
Yes	1705	6.3 ± 2.2	6.0	1.2–18.7	

Testosterone levels are expressed in nmol/L.

**Table 3:** Total and bioavailable testosterone levels determined in different months.

	*N*	Mean^*∗*^	Median	*P* value^*∗*^
*Total testosterone, nmol/L*				
March	2334	14.7 ± 5.5	13.8	
August	2130	15.7 ± 5.9	14.9	<0.001
September	1761	15.7 ± 5.9	15.1	<0.001
October	2093	15.4 ± 5.8	14.7	<0.001

*Bioavailable testosterone, nmol/L*				
March	734	6.7 ± 2.5	6.2	
August	660	7.2 ± 2.8	6.7	<0.001
September	486	7.0 ± 2.8	6.6	<0.001
October	589	7.3 ± 3.0	6.8	<0.001

^*∗*^Values are expressed as mean ± SD. *P* value is derived in comparison with levels in March.

**Table 4:** Linear regression analysis of variables associated with total testosterone (A) and bioavailable testosterone (B).

Variable	*β*	95% CI	*P* value
*(A)*			
Test performed in March vs. August-October	−0.784	−1.046 to −0.523	<0.001
Age (per year)	−0.028	−0.037 to −0.019	<0.001
BMI (per unit)	−0.395	−0.420 to −0.370	<0.001
Current smoking	0.765	0.483 to 1.046	<0.001
Past smoking	0.105	−0.246 to 0.456	0.558
Diabetes	−0.800	−1.170 to −0.431	<0.001

*(B)*			
Test performed in March vs. August–October	−0.593	−1.058 to −0.128	0.013
Age (per year)	−0.029	−0.045 to −0.012	0.001
BMI (per unit)	−0.440	−0.486 to −0.394	<0.001
Current smoking	0.733	0.216 to 1.250	0.005
Past smoking	−0.096	−0.729 to 0.536	0.766
Diabetes	−0.803	−1.461 to −0.145	0.017

## Data Availability

The data used to support the findings of this study are included within the article.
